# Spatial niche partitioning may promote coexistence of *Pygoscelis* penguins as climate‐induced sympatry occurs

**DOI:** 10.1002/ece3.4445

**Published:** 2018-09-11

**Authors:** Erin P. Pickett, William R. Fraser, Donna L. Patterson‐Fraser, Megan A. Cimino, Leigh G. Torres, Ari S. Friedlaender

**Affiliations:** ^1^ Department of Fisheries and Wildlife Marine Mammal Institute Oregon State University Newport Oregon; ^2^ Polar Oceans Research Group Sheridan Montana; ^3^ Scripps Institution of Oceanography University of California San Diego California; ^4^ Institute for Marine Science University of California Santa Cruz Santa Cruz California

**Keywords:** Adélie penguin, climate change, ecological segregation, foraging, gentoo penguin, interspecific competition, *Pygoscelis adeliae*, *Pygoscelis papua*, range shift, space use

## Abstract

Climate‐induced range overlap can result in novel interactions between similar species and potentially lead to competitive exclusion. The West Antarctic Peninsula (WAP) is one of the most rapidly warming regions on Earth and is experiencing a poleward climate migration from a polar to subpolar environment. This has resulted in a range expansion of the ice‐intolerant gentoo penguins (*Pygoscelis papua*) and a coincident decrease in ice‐obligate Adélie penguins (*P. adeliae*) near Palmer Station, Anvers Island, WAP. Ecologically similar species that share a limited prey resource must occupy disparate foraging niches in order to co‐exist. Therefore, we determined the extent of foraging and dietary niche segregation between Adélie and gentoo penguins during the austral breeding season near Palmer Station. This research was conducted across six breeding seasons, from 2009 to 2014, which allowed us to investigate niche overlap in the context of interannual resource variability. Using biotelemetry and diet sampling, we found substantial overlap in the diets of Adélie and gentoo penguins, who primarily consumed Antarctic krill (*Euphausia superba*); however, our results showed that Adélie and gentoo penguins partitioned this shared prey resource through horizontal segregation of their core foraging areas. We did not find evidence that Antarctic krill were a limiting resource during the breeding season or that climate‐induced sympatry of Adélie and gentoo penguins resulted in competition for prey or caused the subsequent differing population trajectories. This apparent absence of resource competition between Adélie and gentoo penguins throughout this study implies that current population trends in this region are governed by other biological and physical factors. Our results highlight the importance of understanding the mechanistic processes that influence top predator populations in the context of climate‐driven ecosystem shifts.

## 1 INTRODUCTION

Interactions between species play a vital role in determining their distribution and community structure and have become increasingly important to understand in the context of climate change (Urban et al., 2016; Zarnetske, Skelly, & Urban, 2012). By altering the physical environment, climate change directly affects species physiology, phenology, and distribution (Hughes, 2000; Walther et al., 2002). When trophically interacting species display differential responses to climate change (e.g., dispersal, extinction, or adaptation), altered biotic interactions can ensue (Durant, Hjermann, Ottersen, & Stenseth, 2007; Schweiger, Settele, Kudrna, Klotz, & Kühn, 2008). For example, climate‐induced range shifts introduce the potential for novel or increased competition between species whose ranges did not historically overlap (Sinervo et al., 2010). Climate‐induced range shifts toward higher elevations and higher latitudes have been observed in a wide variety of taxa including plants, butterflies, birds, and mammals (Walther et al., 2002). These range shifts have been primarily due to increasing temperatures and have disproportionally affected mountaintop and polar species that already exist near the edge of their range limits (Parmesan, 2006). In addition, the effects of climate change are particularly pronounced in Polar Regions, where many species have evolved life history strategies that rely on sea ice (Moline et al., 2008).

Key life history stages of ice‐obligate (i.e., polar) species have been disrupted as a result of warming‐induced habitat shifts and altered trophic interactions, mediated largely by sea ice loss (Moline et al., 2008). Climate warming on the West Antarctic Peninsula (WAP) has occurred at a rapid rate, with midwinter atmospheric temperatures increasing by as much as 6°C over the past 50 years (Vaughan et al., 2003). Combined with rising ocean temperatures, this warming has shortened the annual duration of winter sea ice coverage along the WAP, resulting in a sea ice season that is now on average three months shorter than it was in 1980 (Stammerjohn, Massom, Rind, & Martinson, 2012). A notable consequence of climate change on the WAP has been a shift in the community structure of *Pygoscelis* penguins. Ice‐obligate Adélie penguins (*Pygoscelis adeliae*) have decreased significantly at nearly all breeding sites on the WAP, while ice‐intolerant populations of gentoo penguins (*P. papua*) have increased, especially at new breeding sites in their recently expanded southern range (Lynch, Naveen, Trathan, & Fagan, 2012). While differing tolerances for sea ice may explain penguin distribution shifts, integrating species interactions in the context of these physical drivers has proven challenging (Ducklow et al., 2007; Fraser, Trivelpiece, Ainley, & Trivelpiece, 1992).

A recent hypothesis by Trivelpiece et al. (2011) postulated that penguin population decreases are the result of increased competition between other krill predators and a large‐scale decrease in krill stocks. Ecological niche theory suggests that species with similar requirements cannot co‐exist in a resource‐limited system unless they differ to some degree in how they utilize shared resources (Gause, 1934; Hutchinson, 1958). Inadequate niche segregation leads to niche displacement and competitive exclusion, whereby one species outcompetes another species for a shared resource (Gause, 1934; Schoener, 1983). Competitive exclusion has been well documented through studies of invasive species; however, less is known about the outcome of species interactions following climate‐induced range overlap (Mooney & Cleland, 2001).

The southward expansion of gentoo penguin colonies on the WAP and the recent colonization of a gentoo penguin colony in the Palmer area have introduced the potential for novel foraging range overlap with Adélie penguins breeding in this area. As central‐place foragers, Adélie and gentoo penguins are limited by the availability of resources within their respective foraging ranges and are vulnerable to increases in the number of predators foraging within those areas who share the same prey. Species commonly avoid interspecific competition by differing in their spatiotemporal distributions and diets (Connell, 1961; MacArthur, 1958). Likewise, seabirds often exhibit colony‐specific foraging areas, which alleviate resource competition among conspecifics between neighboring colonies (Cairns, 1989) and, ideally, minimize travel distance to preferred foraging areas (Ashmole, 1963). These segregation mechanisms minimize niche overlap, facilitate resource partitioning, and promote stable coexistence among neighboring populations.

The few studies that have investigated foraging behavior and niche separation of sympatrically breeding Adélie and gentoo penguins (Cimino, Moline, Fraser, Patterson‐Fraser, & Oliver, 2016a; Trivelpiece, Trivelpiece, & Volkman, 1987; Wilson, 2010) showed these species partition prey resources in space and time. Differences in both diel foraging patterns and breeding chronology have been suggested as potential mechanisms of resource partitioning between *Pygoscelis* species (Trivelpiece et al., 1987; Wilson, 2010). In studies of mixed‐species colonies, spatial separation was due largely to differences in dive depth and foraging distance from shore, with Adélie penguins generally foraging farther away from colonies and at shallower depths than gentoo penguins (Cimino, Moline et al., 2016a; Trivelpiece et al., 1987; Wilson, 2010). Cimino, Moline et al. (2016a) showed similar differences between species with additional spatial segregation possibly due to colony‐specific effects, although such effects were not directly addressed. None of these studies included interannual comparisons of Adélie and gentoo penguin foraging niches, although the diets and foraging strategies of both species may vary from year to year.

Around the WAP, Antarctic krill population structure is a primary determinant of penguin dive behavior, diets, and foraging effort during the breeding season (Fraser & Hofmann, 2003; Miller, Karnovsky, & Trivelpiece, 2009; Miller & Trivelpiece, 2008). Antarctic krill display high interannual variability in recruitment strength with cyclical patterns of strong krill recruitment occurring every 4–5 years following episodic increases of phytoplankton production (Fraser & Hofmann, 2003; Quetin & Ross, 2003; Saba et al., 2014; Steinberg et al., 2015). This life history strategy is thought to allow krill, whose life span is 5–6 years, to sustain stable populations despite high environmental variability (Fraser & Hofmann, 2003; Siegel, 2005). In addition to environmental factors such as wind and tidal cycles (Bernard et al., 2017; Oliver et al., 2013), the availability of krill to penguins during the breeding season is influenced by the strength of krill recruitment events, which affect the abundance of krill within penguin foraging ranges (Steinberg et al., 2015). Prior studies have found that penguins alter their diets, dive behavior, and foraging effort in response to the annual population structure of Antarctic krill (Fraser & Hofmann, 2003; Miller & Trivelpiece, 2008; Miller et al., 2009). In the context of foraging niche segregation, the ability of penguins to adapt to changes in prey availability by altering their foraging behavior draws into question whether the extent of interspecific foraging niche segregation observed in one breeding season is consistent across multiple seasons. This is important because population trends of currently stable Antarctic krill stocks near Palmer Station (Saba et al., 2014; Steinberg et al., 2015) may mirror decreases observed farther north as climate warming and sea ice loss continue.

The objective of this study was to determine the extent and consistency of foraging and dietary niche segregation between Adélie and gentoo penguins during the breeding season near Palmer Station, Anvers Island, across six breeding seasons (2009–2014). Building on a recent study by Cimino, Moline et al. (2016a), which found spatial segregation of Adélie and gentoo penguin foraging areas during the 2010–11 breeding season near Palmer Station, we determined whether these penguins’ space‐use patterns and diets were consistent from year to year as the population structure and availability of Antarctic krill varied. Data were collected during the chick‐rearing phase of the penguins’ breeding cycle when the prey demand of both species is highest, and thus, the potential influence of competition was the greatest. Penguin stomach samples were obtained to investigate dietary differences between species and across years and to determine krill size class structure. We examined satellite telemetry and dive data to investigate the extent of horizontal and vertical separation of penguin foraging areas. We predict 1) extensive overlap in the type of prey consumed by Adélie and gentoo penguins, and 2) overlap of prey type will be mitigated by interspecific differences in vertical space use (i.e., dive depth) and prey size. Optimal foraging theory predicts that seabirds will minimize foraging distance from colonies. Thus, due to the distance between the primary study colonies, we expect 3) spatial segregation between Adélie and gentoo penguin foraging areas with minimal overlap at the equidistant line between the primary study colonies.

## 2 MATERIALS AND METHODS

### 2.1 Study site and species

Our study is part of a long‐term monitoring program of Adélie and gentoo penguins breeding in the vicinity of Palmer Station, on Anvers Island, Antarctica. The marine environment in this area is especially productive due to the nearby Palmer Deep submarine canyon (Ducklow et al., 2007; Schofield et al., 2013) that cuts across the continental shelf shoreward from the west and allows for the passage of warm, upper circumpolar deep water from the Antarctic Circumpolar Current toward the southern coast of Anvers Island (Schofield et al., 2013). This nutrient‐rich water mass promotes primary production during the austral summer months, thus supporting large krill stocks and a multitude of krill predators such as seabirds, seals, and whales (Ducklow et al., 2007). During the austral summer, Adélie and gentoo penguins nest on several small, rocky islands in the vicinity of Palmer Station. Our fieldwork occurred on Biscoe Point (64°48′S, 63°46′W), where Adélie and gentoo penguins nest in mostly separate colonies, and Torgersen (64°46′S, 64°04′W) and Humble (64°45′S, 64°05′W) Islands, which are located approximately 15 km to the west of Biscoe Point and occupied solely by Adélie penguins. Humble, and later Torgersen Island, was chosen as Adélie penguin study colonies because they represented the largest of the few remaining colonies in the Palmer area, where colony extinctions have occurred at an alarming rate (Fraser, Patterson‐Fraser, Ribic, Schofield, & Ducklow, 2013). The Adélie penguin breeding population in the Palmer area has decreased by nearly 90% over the past four decades and the most recent population census in 2014 reported a total of 1,976 breeding pairs, with 613 pairs breeding on Humble Island and 1,234 pairs breeding on Torgersen Island (Ducklow et al., 2007; W. Fraser, unpublished data). In comparison, Gentoo penguins first established a colony on Biscoe Point in 1993 and have steadily increased (Bestelmeyer et al., 2011; Ducklow et al., 2007). A total of 3,571 gentoo and 486 Adelie penguin pairs were reported breeding at Biscoe Point in 2014 (W. Fraser, unpublished data). Our fieldwork occurred during the chick‐rearing phase of the penguin breeding season, from the end of December through mid‐February between 2009 (2009–10 field season) and 2014 (2014–15 field season) (Supporting Information Figure S1).

### 2.2 Dietary analysis

In order to evaluate the extent and consistency of dietary niche partitioning between Adélie and gentoo penguins, we collected stomach samples and tested for differences in the type of prey items and the size of Antarctic krill consumed by both species. To gain an accurate representation of penguin diets throughout each season, diet samples were collected roughly every five days (depending on weather conditions) from five individuals of each species per sampling day. Stomach contents were obtained from presumed breeding adults using a water off‐loading method (Wilson, 1984). Birds which appeared to be returning to a nest (i.e., headed from the water's edge to the colony) were captured with a hand net. Diet samples were then collected and animals were subsequently measured, marked, and released back into the colony. Bird gender was determined in the field based on an assessment of head size, bill length, bill depth, and body mass, and we attempted to sample a roughly even ratio of males and females each season (see Supporting Information Table S1 for sex breakdown by year). We collected a total of 136 diet samples from Adélie penguins (77 male, 59 female) and 128 diet samples from gentoo penguins (64 male, 64 female).

Prey samples were drained, sorted, and weighed according to methods outlined by the Commission for the Conservation of Antarctic Marine Living Resources (CCAMLR, 2014). In order to determine diet composition, we identified common prey items and categorized them as either Antarctic krill (*Euphausia superba*), big‐eyed krill (*Thysanoessa macrura*), fish, or “other,” and calculated the proportion of each of these prey items by weight (% wet mass). We recorded the frequency of occurrence of these prey items and noted evidence (e.g., otoliths and bones) of more quickly digested, soft‐bodied prey such as fish. We compared the composition and frequency occurrence of prey between species, by sex and across years using generalized linear models (GLM). GLMs allowed us to account for response variables with non‐normally distributed error and/or heteroscedasticity (Crawley, 2007). Prior to analysis, proportional diet composition data were arcsine‐transformed to achieve normality. We used the “glm” function in the R package lme4 (Bates, Maechler, & Bolker, 2015; R Development Core Team, 2014) with a normal error distribution and identity link function to compare diet composition and a binomial distribution and logit link function to test for differences in frequency of occurrence. The significance of the effect of species on diet composition and frequency of occurrence was assessed using a likelihood ratio test.

In addition to prey type, we investigated the extent of dietary niche partitioning in terms of the size of Antarctic krill (hereafter referred to as “krill”), and the main prey species consumed by Adélie and gentoo penguins. During stomach lavage, fresher, more intact prey is typically regurgitated first, followed by more digested layers of prey. A subsample of roughly 50–100 intact krill was randomly selected from the fresher portion of each diet sample. We measured each of these subsampled krill from the leading edge of the eye to the tip of the telson to obtain total length and binned them into eight size classes in 5‐mm increments from 16 to 65 mm. This binning resolves interannual changes in krill size class structure, as krill grow >5 mm per year (Siegel, 1987) and it allowed us to compare the krill population structure between species and across years. We combined the two smallest size classes (16–20 mm and 21–25 mm) and the two largest size classes (51–55 mm and 56–65 mm) due to low sample size in those bins. We calculated the proportion of krill that fell into each of eight size classes and created size class frequency distributions for each species per year. We tested for differences in size class distributions between species each year using a Pearson's chi‐squared test. We tested for differences in mean krill length between species each year using linear mixed‐effects models (LMM) with bird identity included as a random effect.

### 2.3 Instrumentation and data processing

We conducted a tracking study in conjunction with diet sampling in order to gain a complete picture of the foraging niches of Adélie and gentoo penguins during the breeding season. Instrumentation occurred during roughly the same time as our diet study on an independent sample of breeding adults (Supporting Information Figure S1). We selected penguins for tagging if they were paired and had a brood‐stage nest containing two chicks. Following established methods, we only selected birds for tagging if both birds were present at the nest so that we could compare morphometric differences and assign genders on site (Fraser & Hofmann, 2003). If birds were too close in size and we could not assign a gender, neither bird was tagged. Only one individual from each pair was outfitted with tags, and both sexes were represented approximately equally throughout the study period to minimize the effects of sex‐based differences in foraging areas and/or dive behavior. In total, our tracking effort amounted to 61 Adélie penguins (30 male, 31 female) and 48 gentoo penguins (25 male, 23 female). See Supporting Information Table S1 for sex breakdown by year. We outfitted each penguin with a continuously transmitting ARGOS satellite tag (Sirtrack Limited, Havelock North, New Zealand: KiwiSat202; or Wildlife Computers Redmond, WA, USA: SPOT3, SPOT‐275A, SPOT‐275B, or custom mold based on a SPLASH tag configuration) and a time‐depth recorder (TDR; Lotek Wireless, Inc, St. John's Canada: Lotek LAT1400) that sampled every 1–2s (resolution of 0.05 m and accuracy of ±1 m). See Supporting Information Table S1 for further details on tag specifications. For all years except 2010, TDRs were programmed to begin recording at 5 m, and thus, we were unable to perform a zero offset correction of depth to account for potential drift in pressure transducers. Transmitters and TDRs were fastened to feathers on the lower dorsal region using waterproof Tesa^®^ tape and small plastic zip ties (Wilson & Wilson, 1989). This attachment method minimized drag and allowed for easy tag removal without damaging feathers (Bannasch, Wilson, & Culik, 1994; Wilson & Wilson, 1989). Transmitters remained on penguins for a short duration of time (4.06 ± 1.41 days) and the tags used in our study represented <2% of the body mass of the lightest penguins that are typically captured as part of the Palmer long‐term monitoring program (Adélie range: 3.4–5.4 kg and gentoo range: 4.2–7.4 kg). We did not test for a “device effect” in our study; however, we used the most light‐weight, streamlined transmitters available. We ensured animal welfare using devices whose mass fell well within the “3%” and “5%” rules many biologging studies adhere to and deploying transmitters on individuals for a short duration of time (Kenward, 2001; Phillips, Xavier, & Croxall, 2003). By introducing additional drag on swimming and flying animals, biologgers have been shown to negatively affect foraging efficiency, thus biasing measurements of foraging parameters such as trip duration and dive depth (Ludynia et al., 2012; Ropert‐Coudert, Knott, Chiaradia, & Kato, 2007; Ropert‐Coudert et al.,^2000^); however, in some cases, external devices have not had significant effects on foraging trip duration (Ballard, Ainley, Ribic, & Barton, 2001; Lescroël & Bost, 2005). Nevertheless, we cannot eliminate the possibility of device effects in our study, but because we focused on large‐scale horizontal and vertical space‐use patterns across multiple seasons, rather than fine‐scale foraging parameters, we did not expect device effects to significantly alter the patterns of penguin foraging behavior we observed.

Prior to characterizing penguin space use, we filtered location data following the methods of Oliver et al. (2013) to remove erroneous positions. This involved incorporating ARGOS estimates of positional error (CLS, 2016) into a three‐stage filtering process in which we identified and removed unreasonable locations based on penguin swimming speed (8 km/hr) (Ainley, 2002) and erroneous terrestrial positions that were located on Anvers Island. Because penguins often foraged close to shore among many small, rocky islands, locations found on these smaller islands were not removed. Location data were time matched to dive records and linear interpolation was used to estimate foraging locations for dives that occurred between known locations and within 30 min of a foraging bout. Following the removal of erroneous location data, dive data were then filtered in order to only include dives associated with foraging (hereafter referred to as “forage” dives) following established methods (Cimino, Moline et al., 2016a). This approach allowed us to measure the true extent of each species foraging range and thus the potential for foraging space overlap. We categorized penguin dive types following the methods of Cimino, Moline et al. (2016a) and removed dives that were not foraging dives (e.g., exploratory, or “search” dives and transit dives). Unlike foraging dives, search and transit dives were short in duration (<90 s) and dive profiles lacked evidence of prey pursuit such as plateaus, bottom time and vertical undulations, or “wiggles” (Bost et al., 2007; Chappell, Shoemaker, Janes, Bucher, & Maloney, 1993; Kirkwood & Robertson, 1997; Rodary, Wienecke, & Bost, 2000). For each forage dive, we calculated an estimate of forage depth using a kernel density estimate (Scott, 2015). This estimated foraging depth is the depth where the longest portion of dive time was spent and represents where prey encounter or pursuit was most likely to have occurred.

### 2.4 Spatiotemporal analysis of foraging areas and dive behavior

We created two and three‐dimensional utilization distributions (UD) from known locations of penguin forage dives in order to visualize and quantify the probability distribution of penguin space use. Two‐dimensional kernel density estimation (KDE) techniques allowed us to assess penguin foraging ranges across an *X‐Y* plane, while three‐dimensional KDE incorporated penguin dive depth into our overall assessment of space use. 3D KDE methods have recently been implemented to address the inaccuracies inherent in 2D KDE when depth or height is significant components of foraging ecology (Cooper, Sherry, & Marra, 2014; Simpfendorfer, Olsen, Heupel, & Moland, 2012). We pooled location data by species and by year and used the R package “ks” (Duong, 2007) to calculate KDE of foraging areas. We determined the 50% and 95% KDE of each species spatial distribution and used those isopleths to define “core” and “overall” foraging areas, respectively (Laver & Kelly, 2008). These KDE are commonly used in home range studies to differentiate between core areas of highest use and overall ranges (or territories) (Laver & Kelly, 2008). Data were evaluated using the “ks” default grid resolution of *n* = 151 grid points for 2D estimates and *n* = 51 grid points for 3D estimates. In order to compare pooled datasets with uneven sample sizes, we employed a data‐based “plug‐in” bandwidth selector (“Hpi”), which was calculated for each pooled dataset separately, similar to Gutowsky, Leonard, Conners, Shaffer, and Jonsen (2015). For similar reasons, we used a fixed (vs. local) kernel approach, which applied this smoothing factor consistently across each evaluated point within each dataset (Kie, 2013). We used an iterative subsampling approach to assess the effect of the number of individuals included in our analyses on the core foraging areas of both species to understand whether our limited sample size was a good estimate of foraging range (Gutowsky et al., 2015; Orben et al., 2015; Soanes, Arnould, Dodd, Sumner, & Green, 2013). A visualization of this sample size assessment can be found in the Supplementary Information (Supporting Information Figure S2).

After delineating the foraging ranges of Adélie and gentoo penguins, we quantified the extent of spatial overlap that occurred between the two species foraging areas. We used our 2D and 3D KDE of foraging areas to calculate the proportion of overlap between each species’ overall and core foraging range. This provided us with a simple measure of overlap (“percent overlap”) between the foraging areas of each species for each year. We also employed the Utilization Distribution Overlap Index (UDOI), which provides a single, nondirectional measure of space‐use sharing that accounts for each species’ underlying UD (Cooper et al., 2014; Fieberg & Kochanny, 2005). Because it incorporates the probability distributions of both species foraging ranges, the UDOI complements the more traditional and straightforward percent overlap calculation. UDOI generally varies between 0 and 1, with 0 representing no overlap and 1 indicating complete overlap (Fieberg & Kochanny, 2005). Both calculations were performed in R using code adapted from Fieberg and Kochanny (2005), Simpfendorfer et al. (2012), and Cooper et al. (2014).

To further examine the role of vertical niche partitioning and investigate temporal differences in foraging effort, we compared the forage dive depth and diel patterns of Adélie and gentoo penguin foraging dives. We pooled dives by species and by year and used LMM to test the effect of species on forage dive depth. LMM allowed us to account for individual effects resulting from multiple observations from the same bird. Bird identity was included in the models as a random effect (Faraway, 2016). We pooled data from all seasons and binned foraging dives by hour to assess temporal patterns in foraging effort (i.e., proportion of total dives performed per hour of the day) and foraging depth.

## 3 RESULTS

### 3.1 Dietary overlap

We found evidence of extensive dietary overlap between species and across years, primarily due to the predominance of Antarctic krill in the diet samples. Antarctic krill was present in all of the Adélie and gentoo penguin diet samples and dominated the diets of both species by weight in all years (Table 1). For all years combined, the proportion of Antarctic krill (by wet mass) found in Adélie and gentoo penguin diet samples averaged 92.38% (±22.81) and 97.11% (±10.22) krill. We did not detect any differences in the proportion of Antarctic krill consumed by Adélie and gentoo penguins during any season apart from 2010 (*F*_1,38_ = 6.42, *p* = 0.016). In 2010, another krill species, *T. macrura*, appeared in the diets of both species in significantly higher proportions, with Adélie penguin diets containing more compared to gentoo diets (Table 1). For all years combined, the proportion of *T. macrura* in Adélie and gentoo penguin diet samples averaged 7.24% (±22.84) and 0.79% (±5.51), respectively. We found that gentoo penguins consumed higher proportions of fish than Adélie penguins in 2009 (*F*_1,30_ = 4.11, *p* = 0.052) and 2014 (*F*_1,55_ = 4.68, *p* = 0.035), and fish occurred more frequently in gentoo penguin diets compared to Adélie penguin diets in 2011 ($\chi_{1,48}^{2}$ = 7.47, *p* = 0.006) and 2014 ($\chi_{1,55}^{2}$ = 5.97, *p* = 0.015). For all years combined, the proportion of fish found in Adélie and gentoo penguin diet samples averaged 0.33% (±0.65) and 1.93% (±7.98), respectively. Other prey items found in penguin diets included amphipods, isopods, and mysid species, and were found in low proportions (<1%, Table 1). The proportions and frequency occurrence of these prey items were higher in gentoo penguin diets compared to Adélie penguins in 2009 (*F*_1,31_ = 8.77, *p* = 0.006; $\chi_{1,31}^{2}$ = 7.89, *p* = 0.005) and vice versa in 2010 (*F*_1,38_ = 4.09, *p* = 0.050; $\chi_{1,38}^{2}$ = 6.26, *p* = 0.012) (Table 1). For all years combined, the proportion of “other” prey items found in Adélie and gentoo penguin diet samples averaged 0.04% (±0.18) and 0.17% (±1.13), respectively. We did not find any significant differences in the proportion or frequency occurrence of *E. superba*,* T. macrura*, or fish between sexes for Adélie and gentoo penguins, but did find that the percent contribution of “other” prey items in penguin diets was marginally higher for male Adélies (*F*_1,134_ = 4.20, *p* = 0.042) and female gentoos (*F*_1,126_ = 4.025, *p* = 0.047) and that the frequency occurrence of “other” prey and was higher in the diets of male gentoo penguins ($\chi_{1,126}^{2}$ = 5.42, *p* = 0.020), but did not differ by sex for Adélie penguins ($\chi_{1,134}^{2}$ = 3.12, *p* = 0.078).

**Table 1:** Comparison of Adélie and gentoo penguin diets during the chick‐rearing phase of the breeding season near Palmer Station, Antarctica, from 2009 to 2014. Percent diet composition (mean ± *SD*) is shown with frequency of occurrence (%) of prey items in parentheses. Test statistics and *p*‐values reported for generalized linear models (with those pertaining to frequency occurrence in parentheses). Significant *p*‐values are bolded

Prey type	Year	Adélie	*n*	Gentoo	*n*	Statistic	*p‐*value
*Euphausia superba* (%)	2009	99.2 ± 1.8 (100)	23	98.6 ± 2.6 (100)	10	*F*_1,31_ = 1.28 ($\chi_{1,31}^{2}$ = 0)	0.267 (1)
	2010	63.6 ± 41.0 (100)	25	93.1 ± 15.2 (100)	15	*F*_1,38_ = 6.42 ($\chi_{1,38}^{2}$ = 0)	**0.016** (1)
	2011	99.5 ± 0.6 (100)	15	94.8 ± 16.0 (100)	36	*F*_1,48_ = 2.66 ($\chi_{1,48}^{2}$ = 0)	0.110 (1)
	2012	99.7 ± 0.7 (100)	25	98.5 ± 4.3 (100)	20	*F*_1,43_ = 2.18 ($\chi_{1,43}^{2}$ = 0)	0.147 (1)
	2013	99.4 ± 1.1 (100)	21	99.2 ± 1.7 (100)	18	*F*_1,37_ = 0.06 ($\chi_{1,37}^{2}$ = 0)	0.815 (1)
	2014	97.0 ± 13.0 (100)	27	98.8 ± 2.4 (100)	30	*F*_1,55_ = 0.01 ($\chi_{1,55}^{2}$ = 0)	0.911 (1)
*Thysanoessa macrura* (%)	2009	0.4 ± 1.7 (13.0)	23	0.0 ± 0.0 (0.0)	10	*F*_1,31_ = 0.90 ($\chi_{1,31}^{2}$ = 2.29)	0.350 (0.130)
	2010	36.0 ± 41.0 (56.0)	25	6.6 ± 15.3 (40.0)	15	*F*_1,38_ = 6.38 ($\chi_{1,38}^{2}$ = 0.96)	**0.016** (0.326)
	2011	0.0 ± 0.0 (0.0)	15	0.0 ± 0.0 (0.0)	36	NA	NA
	2012	0.0 ± 0.0 (0.0)	25	0.0 ± 0.0 (0.0)	20	NA	NA
	2013	0.0 ± 0.0 (0.0)	21	0.0 ± 0.0 (0.0)	18	NA	NA
	2014	2.8 ± 13.0 (11.1)	27	0.1 ± 0.4 (3.3)	30	*F*_1,55_ = 1.36 ($\chi_{1,55}^{2}$ = 0.48)	0.249 (0.489)
Unidentified fish (%)	2009	0.4 ± 0.6 (60.9)	23	1.3 ± 2.6 (90.0)	10	*F*_1,30_ = 4.11 ($\chi_{1,31}^{2}$ = 3.19)	**0.052** (0.074)
	2010	0.2 ± 0.3 (40.0)	25	0.3 ± 0.4 (46.7)	15	*F*_1,38_ = 0.65 ($\chi_{1,38}^{2}$ = 0.17)	0.426 (0.680)
	2011	0.4 ± 0.5 (40.0)	15	4.7 ± 14.5 (80.6)	36	*F*_1,48_ = 2.58 ($\chi_{1,48}^{2}$ = 7.47)	0.115 **(0.006)**
	2012	0.2 ± 0.7 (24.0)	25	1.5 ± 4.1 (40.0)	20	*F*_1,43_ = 2.65 ($\chi_{1,43}^{2}$ = 1.32)	0.111 (0.250)
	2013	0.6 ± 1.1 (42.9)	21	0.7 ± 1.4 (27.8)	18	*F*_1,37_ = 0.12 ($\chi_{1,37}^{2}$ = 0.97)	0.726 (0.325)
	2014	0.2 ± 0.5 (22.2)	27	1.1 ± 2.4 (53.3)	30	*F*_1,55_ = 4.68 ($\chi_{1,55}^{2}$ = 5.97)	**0.035 (0.015)**
Other prey items (%)	2009	0.0 ± 0.0 (0.0)	23	0.1 ± 0.1 (30.0)	10	*F*_1,31_ = 8.77 ($\chi_{1,31}^{2}$ = 7.89)	**0.006 (0.005)**
	2010	0.1 ± 0.3 (24.0)	25	0.0 ± 0.0 (0)	15	*F*_1,38_ = 4.09 ($\chi_{1,38}^{2}$ = 6.26)	**0.050 (0.012)**
	2011	0.1 ± 0.3 (13.3)	15	0.5 ± 2.1 (22.2)	36	*F*_1,48_ = 0.68 ($\chi_{1,48}^{2}$ = 0.63)	0.413 (0.427)
	2012	0.0 ± 0.1 (4.0)	25	0.0 ± 0.2 (5.0)	20	*F*_1,43_ = 0.12 ($\chi_{1,43}^{2}$ = 0.03)	0.733 (0.872)
	2013	0.0 ± 0.0 (0.0)	21	0.1 ± 0.4 (5.6)	18	*F*_1,37_ = 1.17 ($\chi_{1,37}^{2}$ = 1.58)	0.286 (0.209)
	2014	0.0 ± 0.0 (3.7)	27	0.0 ± 0.0 (6.7)	30	*F*_1,55_ = 0.05 ($\chi_{1,55}^{2}$ = 0.01)	0.829 (0.940)

**Figure 1:**
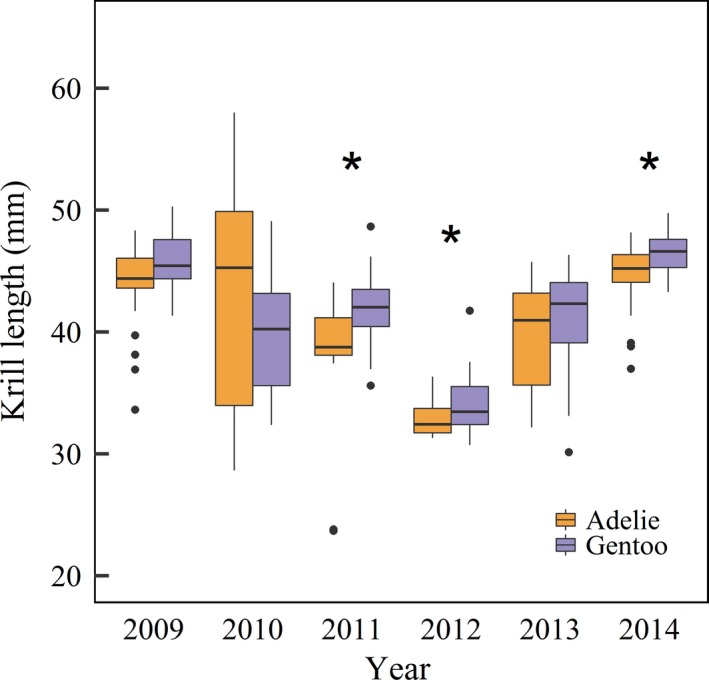
Box plots show the length (mm) of Antarctic krill found in penguin diet samples from 2009 to 2014, and asterisks denote years where significant differences were found using LMM. Boxes represent the 1st quartile, median, and 3rd quartile, and lines indicate minimum and maximum values excluding outliers (points)

**Figure 2:**
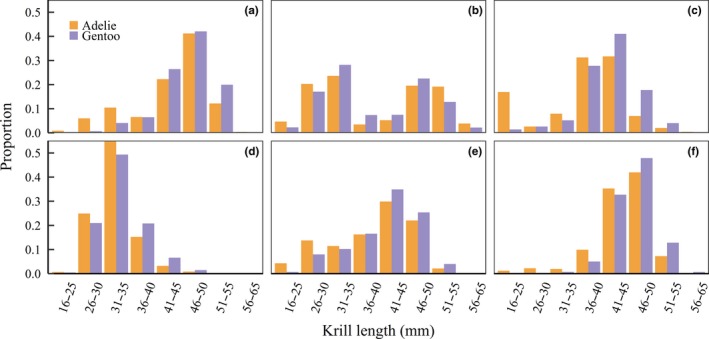
Size class frequency distribution of Antarctic krill in the diets of Adélie and gentoo penguins during the chick‐rearing phase of the breeding season near Palmer Station, Antarctica, from 2009 (a) to 2014 (f)

We found a significant difference in the average size of krill consumed by Adélie and gentoo penguins in 2011 (LMM *t*‐statistic = 3.20, *p* = 0.0025), 2012 (LMM *t*‐statistic = 2.26, *p* = 0.29), and 2014 (LMM *t*‐statistic = 3.19, *p* = .0024), with gentoo penguins consuming larger sized krill than Adélie penguins (Figure 1). We found evidence of species differences in the size class distribution of krill in all years (Table 2), with gentoo penguins consistently selecting significantly larger size classes (Figure 2). Despite differences in the frequency distribution of krill size classes, the dominant size class each year was consistent between species (Figure 2).

**Table 2:** Results of Pearson's chi‐squared tests comparing the size class distribution of Antarctic krill in Adélie and gentoo penguin diet samples from 2009 to 2014

Year	Chi‐squared	*df*	*p*‐value
2009	57.00	8	<0.001
2010	376.80	8	<0.001
2011	269.78	8	<0.001
2012	47.10	8	<0.001
2013	49.14	8	<0.001
2014	151.20	8	<0.001

### 3.2 Spatial segregation of foraging areas

We tracked an average of eight gentoo penguins and 10 Adélie penguins each season. Based on subsampling routines, we found that we tagged a sufficient number of individuals each season, except in 2013, to gain an accurate representation of horizontal space use (Supporting Information Figure S2). It is unlikely that we would have observed a substantial increase in the core foraging areas of either species had more individuals been tagged (Supporting Information Figure S2). In 2013, a combination of transmitter failure and a low number of tracked individuals (gentoo *n* = 4; Adélie *n* = 6) left us with an insufficient amount of data to make reasonable inferences of foraging areas and this year was excluded from our spatial analyses.

**Figure 3:**
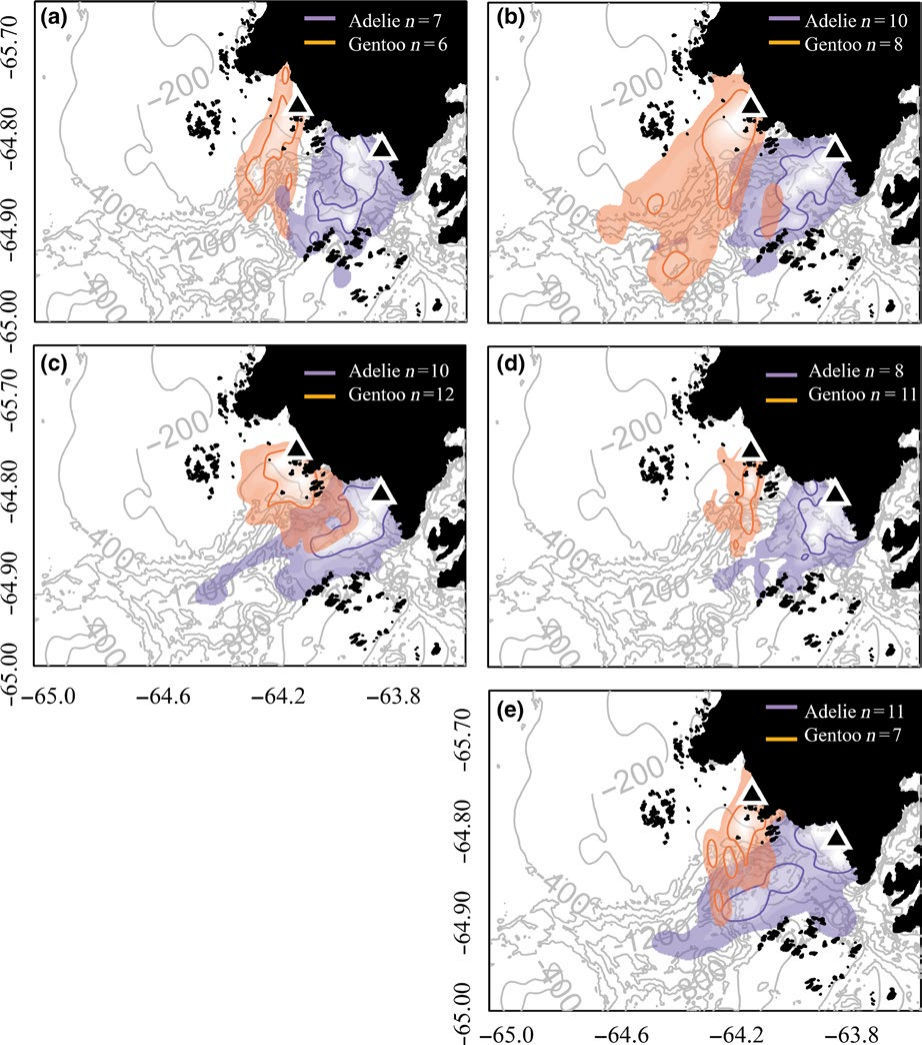
Two‐dimensional foraging areas of Adélie and gentoo penguins during the breeding season near Palmer Station, Antarctica, from 2009 (a) to 2014 (e). Orange shades depict the overall foraging ranges (95% KDE) of Adélie penguins tagged at Humble and Torgersen islands and purple shades depict the overall foraging ranges of gentoo penguins tagged at Biscoe Point. Contour lines outline the core foraging ranges (50% KDE) of both species. Maps produced in R (R Core Team, 2014)

Using 2D and 3D KDE, we found that the foraging effort of Adélie and gentoo penguins was concentrated in separate locations southwest of, and relatively close to, their respective colonies (Figure 3). The 2D core foraging areas (50% KDE) of Adélie and gentoo penguins remained completely separated during every year of the study (Table 3). The 3D core foraging areas remained completely separated during every year of the study apart from 2011, when 1.3% of the Adélie penguin core foraging area overlapped with the gentoo penguin core area (Table 3). For both 2D and 3D KDE, we found minimal overlap near the edges of the overall ranges (95% KDE) of each species in 3 of 4 years (2009–11 & 2014) and no overlap in 2012. The proportion of the 2D Adélie penguin foraging area (95% KDE) that overlapped with that of gentoo penguins ranged from 0.0 to 18.7% (i.e., up to 14.7 km^2^), and the proportion of the gentoo penguin foraging area that overlapped with that of Adélie penguins ranged from 0.0 to 11.2% (i.e., up to 20.9 km^2^) (Table 3). In comparison, the extent of overlap of the foraging areas (95% KDE) of both species was reduced for 3D KDE, ranging from 0.0 to 15.7% for Adélie penguins and 0.0–3.2% for gentoo penguins. We calculated a UDOI value for each overlap comparison (50% and 95% 2D and 3D KDE) between species for each and obtained values ≤0.01 in all cases (Table 3). These UDOI values were consistent with our estimates of percent overlap, suggesting that the probability of spatial overlap between Adélie and gentoo penguin foraging areas was low for all years.

**Table 3:** Overlap indices characterizing spatial overlap of Adélie and gentoo foraging ranges during the chick‐rearing phase of the breeding season near Palmer Station, Antarctica, from 2009 to 2014. Percent overlap and UDOI values shown for 2D KDE with values from 3D KDE in parentheses

Year	Kernel density	Adélie		Gentoo		UDOI
		% overlap with gentoo	*n*	% overlap with Adélie	*n*	
2009	95%	2.2 (1.1)	7	1.3 (0.3)	6	0.00 (0.00)
	50%	0.0 (0.0)		0.0 (0.0)		0.00 (0.00)
2010	95%	4.8 (1.4)	10	4.8 (0.6)	8	0.01 (0.00)
	50%	0.0 (0.0)		0.0 (0.0)		0.00 (0.00)
2011	95%	16.7 (15.7)	10	11.2 (3.2)	12	0.00 (0.01)
	50%	0.0 (1.3)		0.0 (0.3)		0.00 (0.00)
2012	95%	0.0 (0.0)	8	0.0 (0.0)	11	0.00 (0.00)
	50%	0.0 (0.0)		0.0 (0.0)		0.00 (0.00)
2014	95%	18.7 (8.0)	11	7.2 (1.0)	7	0.00 (0.00)
	50%	0.0 (0.0)		0.0 (0.0)		0.00 (0.00)

### 3.3 Dive behavior

**Figure 4:**
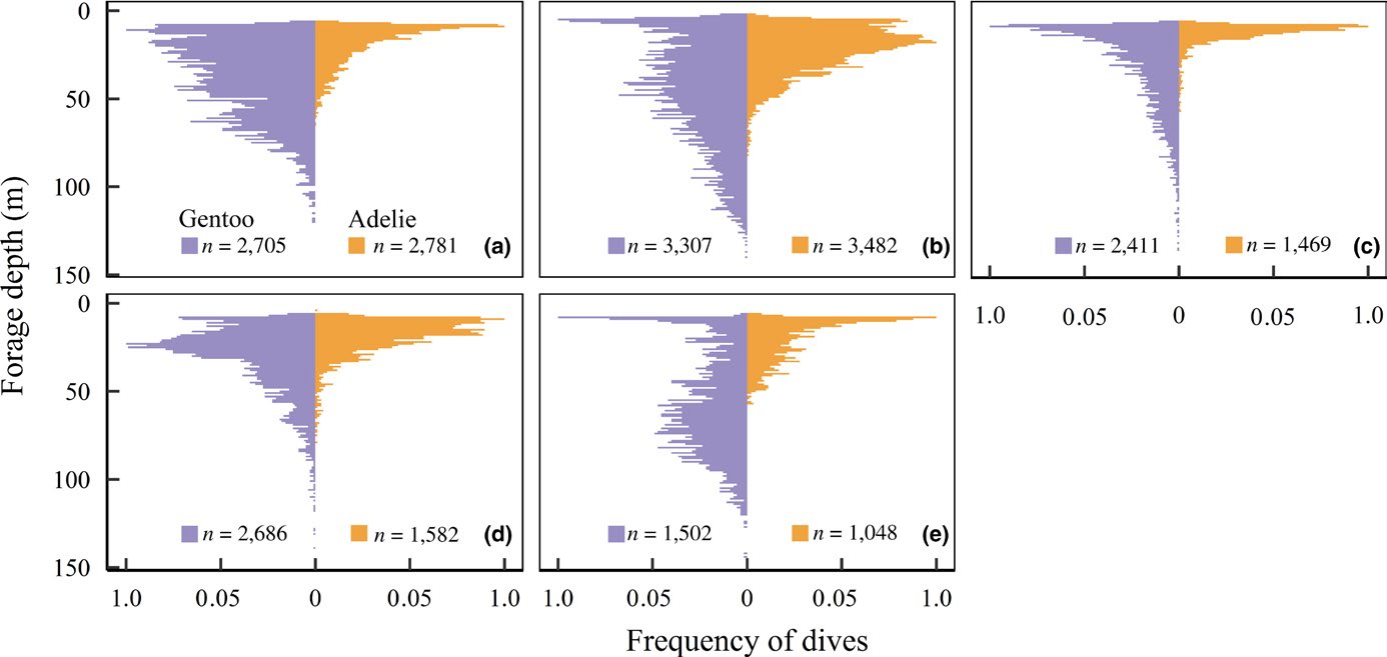
Vertical distribution of Adélie and gentoo penguin foraging dives (binned into 1‐m bins) occurring during the breeding season near Palmer Station, Antarctica, from 2009 (a) to 2014 (e)

**Figure 5:**
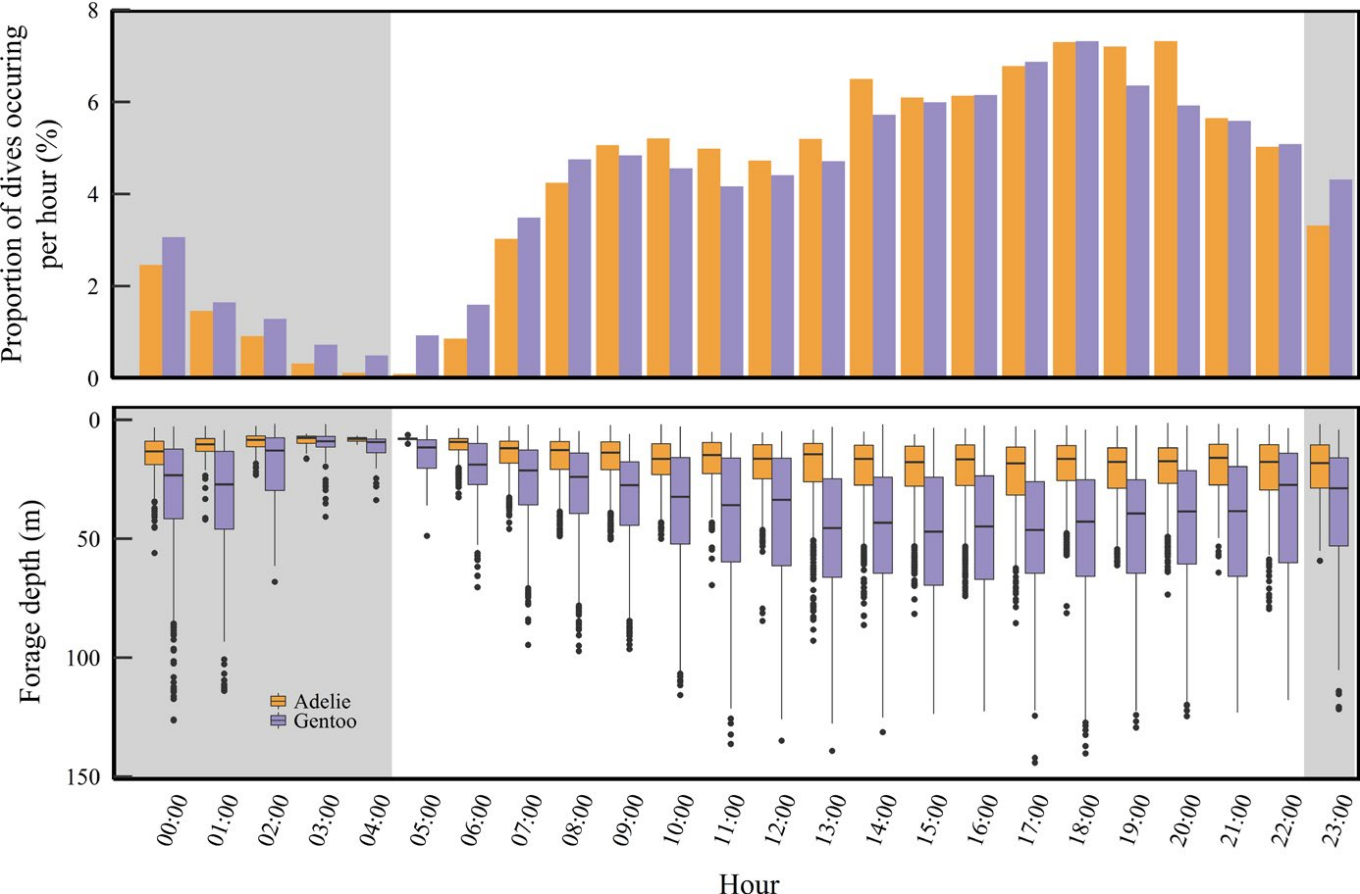
Diel distribution of Adélie and gentoo penguin dive effort (a) and dive depth (b) during the breeding season near Palmer Station, Antarctica, from 2009 to 2014. Shaded rectangles represent mean sunrise and sunset, or nighttime hours during the study period

We found that gentoo penguins utilized a deeper and wider range of depths (average 41.45 ± 23.6 m, range 6–144 m) than Adélie penguins (17.14 ± 8.8 m, range: 6–82 m). Overall, the foraging depth of gentoo penguins was 41.35% deeper than Adélie penguins (LMM χ^2^(1) = 63.29, *p* < 0.001). We found that gentoo penguins dove significantly deeper than Adélie penguins in all years (Table 4, Figure 4). We found that both species generally concentrated their foraging effort during similar daylight hours, with the number of foraging dives occurring per hour increasing throughout the day before peaking around 18:00 local time (Figure 4a). Interestingly, we found the dive depth of both species increased through the day and was the deepest around 15:00; however, this association appeared stronger for gentoo penguins (Figure 5b).

**Table 4:** Depth of Adélie and gentoo penguins foraging dives (mean ± *SD*) and results of LMM that tested the effect of species on foraging dive depth from 2009 to 2014 (excluding 2013)

Year	Foraging depth (m)				$\chi^{2}$	*p*‐value
	*n*	Adélie	*n*	Gentoo		
2009	7	18.3 ± 10.1	6	39.8 ± 22.1	22.65	<0.001
2010	10	23.3 ± 12.1	8	46.7 ± 27.0	13.12	<0.001
2011	10	12.1 ± 5.4	12	35.2 ± 23.6	13.11	<0.001
2012	8	18.1 ± 8.3	11	30.4 ± 17.2	14.91	<0.001
2014	11	13.9 ± 6.8	7	55.1 ± 29.0	45.12	<0.001

## 4 DISCUSSION

In this study, we examined the diets and foraging patterns of Adélie and gentoo penguins in order to gain a better understanding of the ecological implications of climate‐driven range shifts of *Pygoscelis* penguins along the WAP. We found that while both species consistently relied on a similar prey resource (Antarctic krill), Adélie and gentoo penguins partitioned this shared prey resource horizontally by concentrating their foraging effort in separate locations. In some instances, penguins also partitioned the water column vertically by foraging within a different range of depths. We examined these patterns across a complete krill recruitment cycle (6 years) and found that Adélie and gentoo penguins maintained these independent foraging strategies despite variability in the population structure of their primary prey. These results provide a unique multiyear comparison of foraging niche segregation in the context of novel range overlap and build on previous studies that suggest Adélie and gentoo penguins’ exhibit discrete foraging strategies that facilitate resource partitioning in areas of sympatry (Cimino, Moline et al., 2016a; Trivelpiece et al., 1987; Wilson, 2010).

Overall, our results indicate strong dietary overlap between Adélie and gentoo penguins, suggesting that these two species do not partition resources by foraging on different prey during the breeding season. While Antarctic krill was the primary prey of both species, we did observe evidence of other prey items in penguin diets as well as interspecific differences in the size of Antarctic krill consumed by each species. We found that fish occurred more frequently in gentoo penguin diets than in Adélie penguin diets, but even so, fish only represented <2% of the gentoo penguin diet on average. These findings are similar to what has been observed at other breeding sites along the Antarctic Peninsula (Trivelpiece et al., 1987; Volkman, Presler, & Trivelpiece, 1980) and by a recent isotopic diet study conducted near Palmer Station (Gorman, 2015). We also detected small differences in the average size of krill consumed by Adélie and gentoo penguins, whereby gentoo penguins consumed slightly larger krill. Other diet studies have found similar results and differences in prey size have been attributed to differences in beak morphology, energetic requirements, and the spatial distribution of prey in penguin foraging areas (Trivelpiece et al., 1987; Volkman et al., 1980; Wilson, 2010). Fine‐scale data on prey patch composition concurrent with penguin foraging data are necessary in order to determine the ecological significance of these differences in krill size in terms of niche segregation.

We found that the core horizontal foraging areas of Adélie penguins nesting on Humble and Torgersen Islands and gentoo penguins nesting on Biscoe Point were spatially distinct during all years of the study period, similar to the findings of Cimino, Moline et al. (2016a) based on the single 2010 breeding season at Palmer Station. These findings are partially consistent with our predictions that based on optimal foraging theory, and both species would forage according to Cairn's “Hinterland” model (Cairns, 1989) fostering minimal overlap due to physical separation of the primary study colonies. However, the foraging areas that we observed also appear to be influenced by other factors that we did not directly account for in this study. Our study design did not allow us to discern the influence of species versus colony effects on foraging areas, although the foraging areas that we observed are likely driven by density‐dependent effects (see Wakefield et al., 2013), as the nonrandom bearings of Adélie and gentoo penguin foraging areas show that both species generally avoided foraging in the direction of the other's colony (with the exception of Adélie penguins in 2011 & 2014). Future foraging studies should target Adélie penguins from colonies located on Biscoe Point to help elucidate colony‐specific versus species‐specific differences in foraging areas and provide a more complete picture of interspecific niche segregation in this region. It is also important to note that our study did not include an investigation into predator avoidance scenarios, nor physical factors that may concentrate preferred prey patches such as tidal regimes (see Cimino, Moline et al., 2016a & Oliver et al., 2013) or bathymetry, which are all important determinants of foraging behavior. Indeed, the bearings of penguin foraging areas that we observed in this study indicate that the location of the Palmer Deep submarine canyon may influence penguin foraging areas. Nonetheless, we observed clear spatial separation of the core foraging areas of the primary Adélie and gentoo penguin breeding colonies in the Palmer area, which is likely to facilitate interspecific resource partitioning.

A frequently cited mode of niche partitioning by *Pygoscelis* penguins is foraging depth, a dimension that has been likened to tree height in the case of space partitioning by MacArthur's warblers (MacArthur, 1958; Wilson, 2010). We found that gentoo penguins dove deeper on average and exhibited more variable dive depths compared to Adélie penguins. Deeper dives by gentoo penguins have mainly been attributed to their larger body size, with breeding adults weighing 4.9–7.4 kg compared to Adélie penguins, which weigh between 3.6 and 5.5 kg during the chick‐rearing phase (Williams, 1995; Wilson, 2010). For diving animals, physiological constraints that arise due to body size result in optimal foraging depths and habitat specialization (Mori, 2002). Our results indicate that interspecific differences in optimal dive depth may promote vertical resource partitioning between Adélie and gentoo penguins, similar to what has been suggested between humpback and Antarctic minke whales around the Antarctic Peninsula (Friedlaender, Lawson, & Halpin, 2008).

Optimal dive depth is influenced not only by physiological constraints, but also by the vertical distribution and density of prey in the water column (Friedlaender et al., 2016; Hazen, Friedlaender, & Goldbogen, 2015; Mori, 1998). We found interannual differences in penguin dive depth, particularly for gentoo penguins, that may be related to interannual changes in the population structure of Antarctic krill. For example, in 2011, gentoo penguins foraged at much shallower depths compared to 2015 (Figure 4c,e). During that time, penguin diet samples indicated a strong krill recruitment event followed by three successive years where the dominant cohort aged and increased in body size (Figure 2c–f). In addition, we found a diel trend in the foraging depth of gentoo penguins, while the foraging depth of Adélie penguins remained relatively consistent throughout the day and night. These differences may be explained by differing sensitivities to prey density, whereby gentoo penguins are less tolerant than Adélie penguins to sparsely distributed prey. Studies of krill schooling behavior suggest that prey aggregation structure is influenced by diel vertical migration and that denser prey aggregations are located deeper in the water column throughout the day (Zhou & Dorland, 2004). We suspect that gentoo penguins alter their dive depth according to where more profitable, denser prey patches are located. Meanwhile, due to their smaller body size, Adélie penguins may effectively forage in less dense and shallower prey patches (Mori, 2002). Previous research has indicated that differing prey density thresholds facilitate resource partitioning between sympatric seabirds (Ballance, Pitman, & Reilly, 1997; Mori & Boyd, 2004; Piatt, 1990). Thus, differing tolerances to prey density could further facilitate the coexistence of sympatric Adélie and gentoo penguins.

We observed interannual krill recruitment variability in the diets of both Adélie and gentoo penguins, consistent with prior studies that found penguin diets reflect a 4‐ to 5‐year krill recruitment cycle (Fraser & Hofmann, 2003; Miller & Trivelpiece, 2007). Adélie and gentoo penguin foraging strategies (i.e., diet and dive behavior) have been shown to change based on krill size class structure (Fraser & Hofmann, 2003; Lescroël & Bost, 2005; Lynnes, Reid, Croxall, & Trathan, 2002), and thus, variation in foraging locations, depths and/or diets of penguins potentially affects the extent of foraging niche overlap between these two species. Our results indicate that despite variation in krill population structure across six years, Adélie and gentoo penguins continued to rely on krill as a primary prey and maintained spatially separate core foraging areas during the chick‐rearing phase of the breeding season near Palmer Station.

We conducted our tracking and diet studies during the peak of each species respective chick‐rearing period in order to capture penguin foraging patterns during the most energetically demanding stage of the breeding cycle. During our study period, Adélie penguins initiated egg‐laying roughly 2 weeks before gentoo penguins. As a result, data collection was slightly staggered between species, with a focus on Adélie penguins starting and ending earlier in the season. Because breeding phenology affects penguin foraging behavior (e.g., Clarke, Emmerson, & Otahal, 2006; Clarke et al., 1998), the extent of foraging niche overlap that we observed in our study may have differed slightly had data collection overlapped completely between species. Allochrony of *Pygoscelis* penguins may act as an additional dimension of niche partitioning (Trivelpiece et al., 1987) and has been shown to be an important factor driving differences in Adélie and gentoo penguin nesting success under variable weather and climate scenarios (Cimino, Fraser, Patterson‐Fraser, Saba, & Oliver, 2014; Fraser et al., 2013; Hinke et al., 2015). A more detailed investigation into the effects of breeding chronology on Adélie and gentoo penguin foraging niches in this region is warranted and would help to elucidate population‐level responses to rapid environmental change on the WAP.

Resource limitation is a necessary condition for competition to exist (Milne, 1961). The recently revisited krill surplus hypothesis (Laws, 1977; Sladen, 1964; Trivelpiece et al., 2011) cited decreases in krill stocks (Atkinson, Siegel, Pakhomov, & Rothery, 2004) as a driver of declining penguin populations on the WAP, though, a recent assessment of krill stocks along the WAP where our study occurred did not find evidence of a long‐term decrease in Antarctic krill (Steinberg et al., 2015). The recent recovery of Antarctic baleen whales is a focal aspect of Trivelpiece et al.'s (2011) hypothesis that *Pygoscelis* population decreases are the results of increased competition with krill predators; however, populations of blue and fin whales have not rebounded as quickly as humpback whales, leaving overall numbers of large baleen whales still far below historical levels (Branch, Matsuoka, & Miyashita, 2004). Because gentoo penguins rely on krill as a primary prey resource in the Palmer area, declining krill stocks would contradict the rapid increase in gentoo penguin populations. Furthermore, a study of long‐term trends (1989–2011) of Adélie penguin chick fledgling mass (CFM) in the Palmer area determined that Adélie penguin diet characteristics had a minimal influence on CFM, suggesting that penguins had adequate prey resources during the breeding season (Cimino et al., 2014). Despite long‐term population trends indicating that prey resources are not currently limited in the Palmer area, we did not directly measure the distribution and density of prey within our study area. Absolute determinations about the presence or absence of resource competition are often challenging due to the difficulty of obtaining prey data concurrent with predator foraging data and the dynamic nature of the marine environment. For instance, Wilson (2010) found that the mobility of krill may hinder the effectiveness of spatial niche partitioning by *Pygoscelis* penguins if prey patches move between niche hypervolumes. Continued research that incorporates detailed measurements of prey distribution data will enhance our interpretation of the spatial separation we observed between Adélie and gentoo penguin foraging areas. Nonetheless, if prey resources are not limiting in this region, this suggests that competition between penguins during the breeding season is an unlikely driver of local population trends.

To our knowledge, this is the first study to characterize the foraging niches of Adélie and gentoo penguins by assessing horizontal and vertical space utilization and dietary overlap across multiple breeding seasons. In doing so, we investigated two important modes of niche partitioning, diet, and foraging location, and we determined that while these two sympatric predators consistently rely on a shared prey resource, the primary breeding colonies of each species concentrated their foraging effort in spatially separate locations. The timing and location of our study are particularly important due to the rapidly changing environment on the WAP. Our results indicate that during the breeding season, Adélie and gentoo penguins in this area rely heavily on a single prey species that is projected to decline as a result of sea ice loss, though, the results of our tracking study show that adequate spatial separation between foraging areas may buffer against interspecific competition in a resource‐limited scenario. Our results support recent research that has shown other physical mechanisms (e.g., sea ice loss and increased precipitation) and postbreeding season factors (e.g., reduced prey availability) may have a greater influence on the overall success of these two *Pygoscelis* species given future climate projections (Cimino, Lynch, Saba, & Oliver, 2016b; Cimino et al., 2014; Fraser et al., 2013; Hinke, Salwicka, Trivelpiece, Watters, & Trivelpiece, 2007).

## CONFLICT OF INTEREST

None declared.

## AUTHOR CONTRIBUTIONS

W.R.F., A.S.F., and E.P.P. contributed to the development of research questions and methodological design; W.R.F, D.L.P.F., and E.P.P. collected data; M.A.C. and E.P.P. analyzed the data; and all authors contributed to the interpretation of the results and the writing of the manuscript.

## DATA ACCESSIBILITY

All data and related metadata underlying the findings reported in this manuscript will be available online through the Long Term Ecological Research Network Data Portal (https://portal.lternet.edu/nis/home.jsp) within one year of publication.

## References

[ece34445-bib-0001] Ainley, D. (2002). The Adélie penguin: Bellwether of climate change. New York, NY: Columbia University Press 10.7312/ainl12306

[ece34445-bib-0002] Ashmole, N. P. (1963). The regulation of numbers of tropical oceanic birds. Ibis, 103b, 458–473.

[ece34445-bib-0003] Atkinson, A. , Siegel, V. , Pakhomov, E. , & Rothery, P. (2004). Long‐term decline in krill stock and increase in salps within the Southern Ocean. Nature, 432(7013), 100–103. 10.1038/nature02996 15525989

[ece34445-bib-0004] Ballance, L. T. , Pitman, R. L. , & Reilly, S. B. (1997). Seabird community structure along a productivity gradient: Importance of competition and energetic constraint. Ecology, 78(5), 1502–1518. 10.1890/0012-9658(1997)078[1502:SCSAAP]2.0.CO;2

[ece34445-bib-0005] Ballard, G. , Ainley, D. G. , Ribic, C. A. , & Barton, K. R. (2001). Effect of instrument attachment and other factors on foraging trip duration and nesting success of Adélie penguins. The Condor, 103(3), 481–490. 10.1650/0010-5422(2001)103[0481:EOIAAO]2.0.CO;2

[ece34445-bib-0006] Bannasch, R. , Wilson, R. P. , & Culik, B. (1994). Hydrodynamic aspects of design and attachment of a back‐mounted device in penguins. Journal of Experimental Biology, 194(1), 83–96. 9317385 10.1242/jeb.194.1.83

[ece34445-bib-0007] Bates, D. , Maechler, M. , & Bolker, B. (2015). Fitting linear mixed‐effects models using lme4. Journal of Statistical Software, 67(1), 1–48.

[ece34445-bib-0008] Bernard, K. , Cimino, M. , Fraser, W. , Kohut, J. , Oliver, M. , Patterson‐Fraser, D. , … Winsor, P. (2017). Factors that affect the nearshore aggregations of Antarctic krill in a biological hotspot. Deep Sea Research Part I, 127, 139–147. 10.1016/j.dsr.2017.05.008

[ece34445-bib-0009] Bestelmeyer, B. T. , Ellison, A. M. , Fraser, W. R. , Gorman, K. B. , Holbrook, S. J. , Laney, C. M. , … Schmitt, R. J. (2011). Analysis of abrupt transitions in ecological systems. Ecosphere, 2(12), 1–26.

[ece34445-bib-0010] Bost, C. A. , Handrich, Y. , Butler, P. J. , Fahlman, A. , Halsey, L. G. , Woakes, A. J. , & Ropert‐Coudert, Y. (2007). Changes in dive profiles as an indicator of feeding success in king and Adélie penguins. Deep Sea Research Part II: Topical Studies in Oceanography, 54(3), 248–255. 10.1016/j.dsr2.2006.11.007

[ece34445-bib-0011] Branch, T. A. , Matsuoka, K. , & Miyashita, T. (2004). Evidence for increases in Antarctic blue whales based on Bayesian modelling. Marine Mammal Science, 20(4), 726–754. 10.1111/j.1748-7692.2004.tb01190.x

[ece34445-bib-0012] Cairns, D. K. (1989). The regulation of seabird colony size: A hinterland model. The American Naturalist, 134(1), 141–146. 10.1086/284970

[ece34445-bib-0013] CCAMLR (2014). CCAMLR Ecosystem Monitoring Program: Standard methods for monitoring studies. Hobart, Tas, Australia: CCAMLR.

[ece34445-bib-0014] Chappell, M. A. , Shoemaker, V. H. , Janes, D. N. , Bucher, T. L. , & Maloney, S. K. (1993). Diving behavior during foraging in breeding Adélie penguins. Ecology, 74(4), 1204–1215. 10.2307/1940491

[ece34445-bib-0015] Cimino, M. A. , Fraser, W. R. , Patterson‐Fraser, D. L. , Saba, V. S. , & Oliver, M. J. (2014). Large‐scale climate and local weather drive interannual variability in Adélie penguin chick fledging mass. Marine Ecology Progress Series, 513, 253–268. 10.3354/meps10928

[ece34445-bib-0016] Cimino, M. A. , Lynch, H. J. , Saba, V. S. , & Oliver, M. J. (2016b). Projected asymmetric response of Adélie penguins to Antarctic climate change. Scientific Reports, 6: 28785; 10.1038/srep28785 27352849 PMC4926113

[ece34445-bib-0017] Cimino, M. A. , Moline, M. A. , Fraser, W. R. , Patterson‐Fraser, D. L. , & Oliver, M. J. (2016a). Climate‐driven sympatry may not lead to foraging competition between congeneric top‐predators. Scientific Reports, 6: 18820; 10.1038/srep18820 26732496 PMC4702144

[ece34445-bib-0018] Clarke, J. , Emmerson, L. M. , & Otahal, P. (2006). Environmental conditions and life history constraints determine foraging range in breeding Adélie penguins. Marine Ecology Progress Series, 310, 247–261. 10.3354/meps310247

[ece34445-bib-0019] Clarke, J. , Manly, B. , Kerry, K. , Gardner, H. , Franchi, E. , Corsolini, S. , & Focardi, S. (1998). Sex differences in Adélie penguin foraging strategies. Polar Biology, 20(4), 248–258. 10.1007/s003000050301

[ece34445-bib-0020] CLS (Collecte Localisation Satellites) (2016). Argos user's manual. Retrieved from http://www.argos-system.org/manual/

[ece34445-bib-0021] Connell, J. H. (1961). The influence of interspecific competition and other factors on the distribution of the barnacle *Chthamalus stellatus* . Ecology, 42(4), 710–723. 10.2307/1933500

[ece34445-bib-0022] Cooper, N. W. , Sherry, T. W. , & Marra, P. P. (2014). Modeling three‐dimensional space use and overlap in birds. The Auk, 131(4), 681–693. 10.1642/AUK-14-17.1

[ece34445-bib-0023] Crawley, M. J. (2007). Generalized linear models The R book, 1st ed. (pp. 511–526). Wiley Publishing.

[ece34445-bib-0024] Ducklow, H. W. , Baker, K. , Martinson, D. G. , Quetin, L. B. , Ross, R. M. , Smith, R. C. , … Fraser, W. (2007). Marine pelagic ecosystems: The west Antarctic Peninsula. Philosophical Transactions of the Royal Society B: Biological Sciences, 362(1477), 67–94. 10.1098/rstb.2006.1955 PMC1764834 17405208

[ece34445-bib-0025] Duong, T. (2007). ks: Kernel density estimation and kernel discriminant analysis for multivariate data in R. Journal of Statistical Software, 21(7), 1–16.

[ece34445-bib-0026] Durant, J. M. , Hjermann, D. Ø. , Ottersen, G. , & Stenseth, N. C. (2007). Climate and the match or mismatch between predator requirements and resource availability. Climate Research, 33(3), 271–283. 10.3354/cr033271

[ece34445-bib-0027] Faraway, J. J. (2016). Extending the linear model with R: Generalized linear, mixed effects and nonparametric regression models (Vol. 124). Boca Raton, FL: CRC Press.

[ece34445-bib-0028] Fieberg, J. , & Kochanny, C. O. (2005). Quantifying home‐range overlap: The importance of the utilization distribution. Journal of Wildlife Management, 69(4), 1346–1359.

[ece34445-bib-0029] Fraser, W. R. , & Hofmann, E. E. (2003). A predator¹s perspective on causal links between climate change, physical forcing and ecosystem response. Marine Ecology Progress Series, 265, 1–15. 10.3354/meps265001

[ece34445-bib-0030] Fraser, W. R. , Patterson‐Fraser, D. L. , Ribic, C. A. , Schofield, O. , & Ducklow, H. (2013). A nonmarine source of variability in Adélie penguin demography. Oceanography, 26(3), 207–209. 10.5670/oceanog

[ece34445-bib-0031] Fraser, W. R. , Trivelpiece, W. Z. , Ainley, D. G. , & Trivelpiece, S. G. (1992). Increases in Antarctic penguin populations: Reduced competition with whales or a loss of sea ice due to environmental warming? Polar Biology, 11, 525–531.

[ece34445-bib-0032] Friedlaender, A. S. , Johnston, D. W. , Tyson, R. B. , Kaltenberg, A. , Goldbogen, J. A. , Stimpert, A. K. , Curtice, C. , Hazen, E. L. , Halpin, P. N. , Read, A. J. , & Nowacek, D. P. (2016). Multiple‐stage decisions in a marine central‐place forager. Royal Society Open Science, 3(5), 160043. 27293784 10.1098/rsos.160043 PMC4892446

[ece34445-bib-0033] Friedlaender, A. S. , Lawson, G. L. , & Halpin, P. N. (2008). Evidence of resource partitioning between humpback and minke whales in the Antarctic. Marine Mammal Science, 25, 402–415.

[ece34445-bib-0034] Gause, G. F. (1934). Experimental analysis of Vito Volterra's mathematical theory of the struggle for existence. Science, 79(2036), 16–17. 10.1126/science.79.2036.16-a 17821472

[ece34445-bib-0035] Gorman, K. B. (2015). Integrative studies of southern ocean food‐webs and pygoscelis penguin demography: Mechanisms of population response to environmental change. Doctoral Dissertation. Retrieved from http://summit.sfu.ca/item/15610

[ece34445-bib-0036] Gutowsky, S. E. , Leonard, M. L. , Conners, M. G. , Shaffer, S. A. , & Jonsen, I. D. (2015). Individual‐level variation and higher‐level interpretations of space use in wide‐ranging species: An albatross case study of sampling effects. Frontiers in Marine Science, 2, 93.

[ece34445-bib-0037] Hazen, E. L. , Friedlaender, A. S. , & Goldbogen, J. A. (2015). Blue whales (Balaenoptera musculus) optimize foraging efficiency by balancing oxygen use and energy gain as a function of prey density. Science Advances, 1(9), e1500469. 26601290 10.1126/sciadv.1500469 PMC4646804

[ece34445-bib-0038] Hinke, J. T. , Polito, M. J. , Goebel, M. E. , Jarvis, S. , Reiss, C. S. , Thorrold, S. R. , … Watters, G. M. (2015). Spatial and isotopic niche partitioning during winter in chinstrap and Adélie penguins from the South Shetland Islands. Ecosphere, 6(7), 1–32.

[ece34445-bib-0039] Hinke, J. T. , Salwicka, K. , Trivelpiece, S. G. , Watters, G. M. , & Trivelpiece, W. Z. (2007). Divergent responses of Pygoscelis penguins reveal a common environmental driver. Oecologia, 153(4), 845 10.1007/s00442-007-0781-4 17566778

[ece34445-bib-0040] Hughes, L. (2000). Biological consequences of global warming: Is the signal already apparent? Trends in Ecology & Evolution, 15(2), 56–61. 10.1016/S0169-5347(99)01764-4 10652556

[ece34445-bib-0041] Hutchinson, G. E. (1958). Concluding remarks. Cold Spring Harbour Symposium on Quantitative Biology, 22, 417–427.

[ece34445-bib-0042] Kenward, R. E. (2001). A manual for wildlife radio tagging (2nd ed.). San Diego, CA: Academic Press.

[ece34445-bib-0043] Kie, J. G. (2013). A rule‐based ad hoc method for selecting a bandwidth in kernel home‐range analyses. Animal Biotelemetry, 1(1), 13 10.1186/2050-3385-1-13

[ece34445-bib-0044] Kirkwood, R. , & Robertson, G. (1997). The foraging ecology of female emperor penguins in winter. Ecological Monographs, 67(2), 155–176.

[ece34445-bib-0045] Laver, P. N. , & Kelly, M. J. (2008). A critical review of home range studies. Journal of Wildlife Management, 72(1), 290–298. 10.2193/2005-589

[ece34445-bib-0046] Laws, R. M. (1977). Seals and whales of the Southern Ocean. Philosophical Transactions of the Royal Society of London B: Biological Sciences, 279(963), 81–96. 10.1098/rstb.1977.0073

[ece34445-bib-0047] Lescroël, A. , & Bost, C. A. (2005). Foraging under contrasting oceanographic conditions: The gentoo penguin at Kerguelen Archipelago. Marine Ecology Progress Series, 302, 245–261. 10.3354/meps302245

[ece34445-bib-0048] Ludynia, K. , Dehnhard, N. , Poisbleau, M. , Demongin, L. , Masello, J. F. , & Quillfeldt, P. (2012). Evaluating the impact of handling and logger attachment on foraging parameters and physiology in southern rockhopper penguins. PLoS One, 7(11), e50429. 23185623 10.1371/journal.pone.0050429 PMC3503963

[ece34445-bib-0049] Lynch, H. J. , Naveen, R. , Trathan, P. N. , & Fagan, W. F. (2012). Spatially integrated assessment reveals widespread changes in penguin populations on the Antarctic Peninsula. Ecology, 93(6), 1367–1377. 10.1890/11-1588.1 22834377

[ece34445-bib-0050] Lynnes, A. , Reid, K. , Croxall, J. , & Trathan, P. (2002). Conflict or co‐existence? Foraging distribution and competition for prey between Adélie and chinstrap penguins. Marine Biology, 141(6), 1165–1174.

[ece34445-bib-0051] MacArthur, R. H. (1958). Population ecology of some warblers of northeastern coniferous forests. Ecology, 39, 599–619. 10.2307/1931600

[ece34445-bib-0052] Miller, A. K. , Karnovsky, N. J. , & Trivelpiece, W. Z. (2009). Flexible foraging strategies of gentoo penguins *Pygoscelis papua* over 5 years in the South Shetland Islands, Antarctica. Marine Biology, 156(12), 2527–2537. 10.1007/s00227-009-1277-z

[ece34445-bib-0053] Miller, A. K. , & Trivelpiece, W. Z. (2007). Cycles of *Euphausia superba* recruitment evident in the diet of *Pygoscelid* penguins and net trawls in the South Shetland Islands, Antarctica. Polar Biology, 30(12), 1615–1623. 10.1007/s00300-007-0326-7

[ece34445-bib-0054] Miller, A. K. , & Trivelpiece, W. Z. (2008). Chinstrap penguins alter foraging and diving behavior in response to the size of their principle prey, Antarctic krill. Marine Biology, 154(2), 201–208. 10.1007/s00227-008-0909-z

[ece34445-bib-0055] Milne, A. (1961). Definition of competition among animals. Symposia of the Society for Experimental Biology, 15, 40–61.

[ece34445-bib-0056] Moline, M. A. , Karnovsky, N. J. , Brown, Z. , Divoky, G. J. , Frazer, T. K. , Jacoby, C. A. , … Fraser, W. R. (2008). High latitude changes in ice dynamics and their impact on polar marine ecosystems. Annals of the New York Academy of Sciences, 1134(1), 267–319. 10.1196/annals.1439.010 18566098

[ece34445-bib-0057] Mooney, H. A. , & Cleland, E. E. (2001). The evolutionary impact of invasive species. Proceedings of the National Academy of Sciences of the United States of America, 98(10), 5446–5451. 10.1073/pnas.091093398 11344292 PMC33232

[ece34445-bib-0058] Mori, Y. (1998). Optimal choice of foraging depth in divers. Journal of Zoology, 245(3), 279–283. 10.1111/j.1469-7998.1998.tb00102.x

[ece34445-bib-0059] Mori, Y. (2002). Optimal diving behaviour for foraging in relation to body size. Journal of Evolutionary Biology, 15(2), 269–276. 10.1046/j.1420-9101.2002.00382.x

[ece34445-bib-0060] Mori, Y. , & Boyd, I. L. (2004). Segregation of foraging between two sympatric penguin species: Does rate maximisation make the difference? Marine Ecology Progress Series, 275, 241–249. 10.3354/meps275241

[ece34445-bib-0061] Oliver, M. J. , Irwin, A. , Moline, M. A. , Fraser, W. , Patterson, D. , Schofield, O. , & Kohut, J. (2013). Adélie penguin foraging location predicted by tidal regime switching. PloS One, 8(1), e55163. 23383091 10.1371/journal.pone.0055163 PMC3559330

[ece34445-bib-0062] Orben, R. A. , Irons, D. B. , Paredes, R. , Roby, D. D. , Phillips, R. A. , & Shaffer, S. A. (2015). North or south? Niche separation of endemic red‐legged kittiwakes and sympatric black‐legged kittiwakes during their non‐breeding migrations. Journal of Biogeography, 42(2), 401–412. 10.1111/jbi.12425

[ece34445-bib-0063] Parmesan, C. (2006). Ecological and evolutionary responses to recent climate change. Annual Review of Ecology, Evolution, and Systematics, 37, 637–669.

[ece34445-bib-0064] Phillips, R. A. , Xavier, J. C. , & Croxall, J. P. (2003). Effects of satellite transmitters on albatrosses and petrels. The Auk, 120(4), 1082–1090.

[ece34445-bib-0065] Piatt, J. F. (1990). The aggregative response of common murres and Atlantic puffins to schools of capelin. Studies in Avian Biology, 14, 36–51.

[ece34445-bib-0066] Quetin, L. B. , & Ross, R. M. (2003). Episodic recruitment in Antarctic krill *Euphausia superba* in the Palmer LTER study region. Marine Ecology Progress Series, 259, 185–200. 10.3354/meps259185

[ece34445-bib-0067] R Core Team (2014). R: A language and environment for statistical computing. Vienna, Austria: R Foundation for Statistical Computing Retrieved from http://www.R-project.org/

[ece34445-bib-0068] Rodary, D. , Wienecke, B. C. , & Bost, C. A. (2000). Diving behaviour of Adélie penguins (*Pygoscelis adeliae*) at Dumont D'Urville, Antarctica: Nocturnal patterns of diving and rapid adaptations to changes in sea‐ice condition. Polar Biology, 23(2), 113–120. 10.1007/s003000050016

[ece34445-bib-0069] Ropert‐Coudert, Y. , Bost, C. A. , Handrich, Y. , Bevan, R. M. , Butler, P. J. , Woakes, A. J. , & Le Maho, Y. (2000). Impact of externally attached loggers on the diving behaviour of the king penguin. Physiological and Biochemical Zoology, 73(4), 438–444. 10.1086/317743 11009397

[ece34445-bib-0070] Ropert‐Coudert, Y. , Knott, N. , Chiaradia, A. , & Kato, A. (2007). How do different data logger sizes and attachment positions affect the diving behaviour of little penguins? Deep Sea Research Part II: Topical Studies in Oceanography, 54(3–4), 415–423. 10.1016/j.dsr2.2006.11.018

[ece34445-bib-0071] Saba, G. K. , Fraser, W. R. , Saba, V. S. , Iannuzzi, R. A. , Coleman, K. E. , Doney, S. C. , … Stammerjohn, S. E. (2014). Winter and spring controls on the summer food web of the coastal West Antarctic Peninsula. Nature Communications, 5, 4318 10.1038/ncomms5318 25000452

[ece34445-bib-0072] Schoener, T. W. (1983). Field experiments on interspecific competition. American Naturalist, 122, 240–285. 10.1086/284133

[ece34445-bib-0073] Schofield, O. , Ducklow, H. , Bernard, K. , Doney, S. , Patterson‐Fraser, D. , Gorman, K. , … Fraser, W. (2013). Penguin biogeography along the West Antarctic Peninsula: Testing the Canyon Hypothesis with Palmer LTER observations. Oceanography, 26(3), 204–206. 10.5670/oceanog

[ece34445-bib-0074] Schweiger, O. , Settele, J. , Kudrna, O. , Klotz, S. , & Kühn, I. (2008). Climate change can cause spatial mismatch of trophically interacting species. Ecology, 89(12), 3472–3479. 10.1890/07-1748.1 19137952

[ece34445-bib-0075] Scott, D. W. (2015). Multivariate density estimation: Theory, practice, and visualization. London, UK: John Wiley & Sons 10.1002/9781118575574

[ece34445-bib-0076] Siegel, V. (1987). Age and growth of Antarctic *Euphausiacea* (Crustacea) under natural conditions. Marine Biology, 96(4), 483–495. 10.1007/BF00397966

[ece34445-bib-0077] Siegel, V. (2005). Distribution and population dynamics of *Euphausia superba*: Summary of recent findings. Polar Biology, 29(1), 1–22. 10.1007/s00300-005-0058-5

[ece34445-bib-0078] Simpfendorfer, C. A. , Olsen, E. M. , Heupel, M. R. , & Moland, E. (2012). Three‐dimensional kernel utilization distributions improve estimates of space use in aquatic animals. Canadian Journal of Fisheries and Aquatic Sciences, 69(3), 565–572. 10.1139/f2011-179

[ece34445-bib-0079] Sinervo, B. , Mendez‐De‐La‐Cruz, F. , Miles, D. B. , Heulin, B. , Bastiaans, E. , Villagrán‐Santa Cruz, M. , … Gadsden, H. (2010). Erosion of lizard diversity by climate change and altered thermal niches. Science, 328(5980), 894–899. 10.1126/science.1184695 20466932

[ece34445-bib-0080] Sladen, W. J. L. (1964). The distribution of the Adelie and chinstrap penguins In Carrick R., Holdgate M. W., & Prevost J. (Eds.), Biologie Antarctique (pp. 359–365). Paris: Hermann.

[ece34445-bib-0081] Soanes, L. M. , Arnould, J. P. , Dodd, S. G. , Sumner, M. D. , & Green, J. A. (2013). How many seabirds do we need to track to define home‐range area? Journal of Applied Ecology, 50(3), 671–679. 10.1111/1365-2664.12069

[ece34445-bib-0082] Stammerjohn, S. , Massom, R. , Rind, D. , & Martinson, D. (2012). Regions of rapid sea ice change: An inter‐hemispheric seasonal comparison. Geophysical Research Letters, 39(6), L06501.

[ece34445-bib-0083] Steinberg, D. K. , Ruck, K. E. , Gleiber, M. R. , Garzio, L. M. , Cope, J. S. , Bernard, K. S. , … Ross, R. M. (2015). Long‐term (1993–2013) changes in macrozooplankton off the Western Antarctic Peninsula. Deep Sea Research Part I: Oceanographic Research Papers, 101, 54–70. 10.1016/j.dsr.2015.02.009

[ece34445-bib-0084] Trivelpiece, W. Z. , Hinke, J. T. , Miller, A. K. , Reiss, C. S. , Trivelpiece, S. G. , & Watters, G. M. (2011). Variability in krill biomass links harvesting and climate warming to penguin population changes in Antarctica. Proceedings of the National Academy of Sciences of the United States of America, 108(18), 7625–7628. 10.1073/pnas.1016560108 21482793 PMC3088573

[ece34445-bib-0085] Trivelpiece, W. Z. , Trivelpiece, S. G. , & Volkman, N. J. (1987). Ecological segregation of Adélie, gentoo, and chinstrap penguins at King George Island, Antarctica. Ecology, 68(2), 351–361. 10.2307/1939266

[ece34445-bib-0086] Urban, M. C. , Bocedi, G. , Hendry, A. P. , Mihoub, J. B. , Pe'er, G. , Singer, A. , Bridle, J. R. , Crozier, L. G. , De Meester, L. , Godsoe, W. , & Gonzalez, A. , (2016). Improving the forecast for biodiversity under climate change. Science, 353(6304), aad8466 10.1126/science.aad8466 27609898

[ece34445-bib-0087] Vaughan, D. G. , Marshall, G. J. , Connolley, W. M. , Parkinson, C. , Mulvaney, R. , Hodgson, D. A. , … Turner, J. (2003). Recent rapid regional climate warming on the Antarctic Peninsula. Climatic Change, 60(3), 243–274. 10.1023/A:1026021217991

[ece34445-bib-0088] Volkman, N. J. , Presler, P. , & Trivelpiece, W. (1980). Diets of *Pygoscelid* penguins at King George Island, Antarctica. Condor, 82, 373–378. 10.2307/1367558

[ece34445-bib-0089] Wakefield, E. D. , Bodey, T. W. , Bearhop, S. , Blackburn, J. , Colhoun, K. , Davies, R. , … Jessopp, M. J. (2013). Space partitioning without territoriality in gannets. Science, 341(6141), 68–70. 10.1126/science.1236077 23744776

[ece34445-bib-0090] Walther, G. R. , Post, E. , Convey, P. , Menzel, A. , Parmesan, C. , Beebee, T. J. , … Bairlein, F. (2002). Ecological responses to recent climate change. Nature, 416(6879), 389–395. 10.1038/416389a 11919621

[ece34445-bib-0091] Williams, A. (1995). The penguins spheniscidae. Oxford, UK: Oxford University Press.

[ece34445-bib-0092] Wilson, R. (1984). An improved stomach pump for penguins and other seabirds. Journal of Field Ornithology, 55, 110–112.

[ece34445-bib-0093] Wilson, R. P. (2010). Resource partitioning and niche hyper‐volume overlap in free‐living *Pygoscelid* penguins. Functional Ecology, 24(3), 646–657. 10.1111/j.1365-2435.2009.01654.x

[ece34445-bib-0094] Wilson, R. P. , & Wilson, M. P. T. (1989). Tape: A package‐attachment technique for penguins. Wildlife Society Bulletin, 17, 77–79.

[ece34445-bib-0095] Zarnetske, P. L. , Skelly, D. K. , & Urban, M. C. (2012). Biotic multipliers of climate change. Science, 336(6088), 1516–1518. 10.1126/science.1222732 22723403

[ece34445-bib-0096] Zhou, M. , & Dorland, R. D. (2004). Aggregation and vertical migration behavior of Euphausia superba. Deep Sea Research Part II: Topical Studies in Oceanography, 51(17), 2119–2137. 10.1016/j.dsr2.2004.07.009

