# Supplementary material for: Spatial niche partitioning may promote coexistence of Pygoscelis penguins as climate‐induced sympatry occurs
Source: Ecol Evol. 2018 Sep 11;8(19):9764–78. doi: 10.1002/ece3.4445 (PMC6202752; doi:10.1002/ece3.4445)
Supplement: Supplementary file 1 [file ECE3-8-9764-s001.docx]

Supplementary Information

Fig. S1. Timetable of penguin field studies at Palmer Station, Antarctica, from 2009-2014. Asterisks indicate 2013 tracking data that were not used in spatial analyses. Generally, Adélie penguins were targeted earlier than gentoo penguins each season so that tagging and diet sampling occurred during the peak of each species respective chick rearing period.


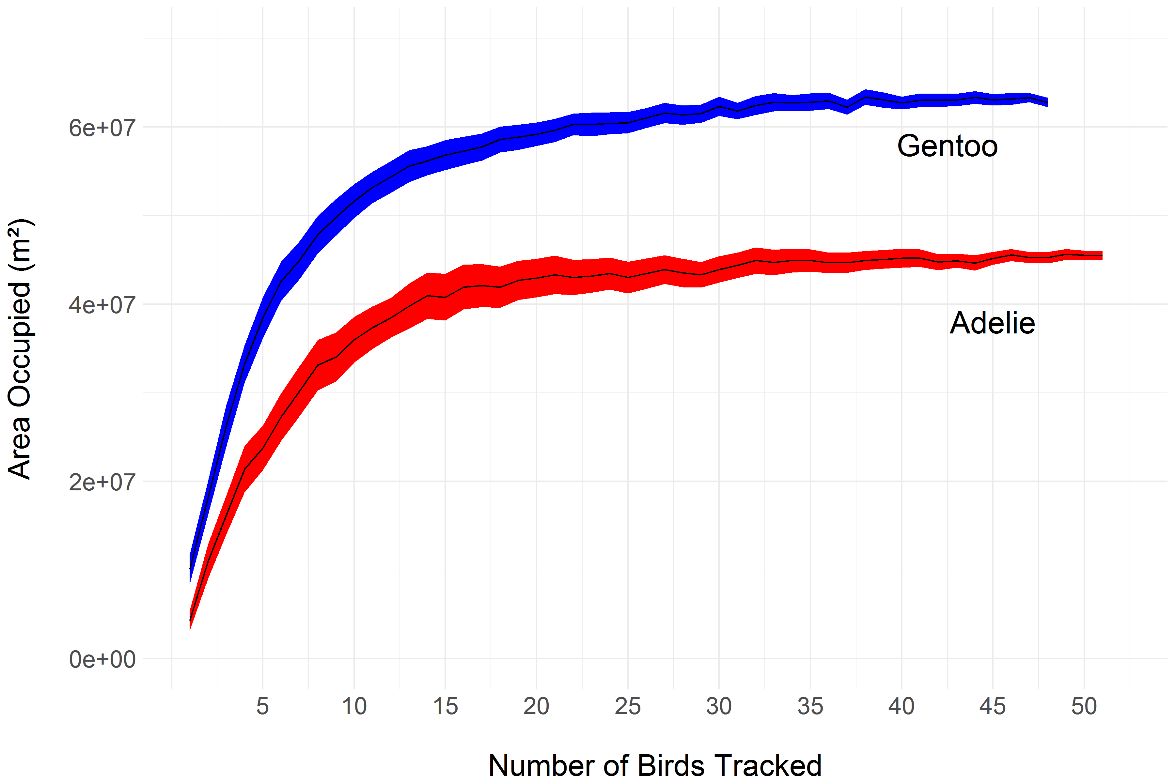


Fig. S2. Asymptotic curves display cumulative core foraging area (m²) relative to the number of individual birds tracked. Bands represent the 95% confidence interval of total core foraging area (50% KDE) occupied by Adélie (in red) and gentoo penguins (in blue).

| **Year** | **Species** | **Island** | ***n* (diet sampled)** | | | ***n* (instrumented individuals)** | | | **TDR manufacturer & model** | **Satellite tag manufacturer & model** |
| --- | --- | --- | --- | --- | --- | --- | --- | --- | --- | --- |
|  |  |  | ***Total*** | **Female** | **Male** | ***Total*** | **Female** | **Male** |  |  |
| 2009 | ADPE | HUM | *23* | 12 | 11 | *7* | 3 | 4 | Lotek Wireless Inc., LAT1400 (11x35mm, 5.2g) | Sirtrack Ltd., KiwiSat 202 (60x27x17mm, 32g) |
|  | GEPE | BIS | *10* | 4 | 6 | *6* | 3 | 3 |  |  |
| 2010 | ADPE | HUM | *25* | 13 | 12 | *10* | 6 | 4 | Lotek Wireless Inc., LAT1400 (11x35mm, 5.2g) | Wildlife Computers, custom mold based on SPLASH config. (86x17x18mm, 55g) |
|  | GEPE | BIS | *15* | 8 | 7 | *8* | 5 | 3 |  |  |
| 2011 | ADPE | HUM | *15* | 7 | 8 | *10* | 6 | 4 | Lotek Wireless Inc., LAT1400 (11x35mm, 5.2g) | Wildlife Computers Inc., SPOT3 (89x15x24mm, 52g) |
|  | GEPE | BIS | *35* | 20 | 15 | *12* | 6 | 6 |  |  |
| 2012 | ADPE | TOR | *25* | 11 | 14 | *8* | 4 | 4 | Lotek Wireless Inc., LAT1400 (11x35mm, 5.2g) | Wildlife Computers Inc., SPOT275 (86x17x18mm, 35g) |
|  | GEPE | BIS | *20* | 11 | 9 | *11* | 4 | 7 |  |  |
| 2013 | ADPE | TOR | *21* | 7 | 14 | *-* | - | - | - | - |
|  | GEPE | BIS | *18* | 6 | 12 | *-* | - | - |  |  |
| 2014 | ADPE | TOR | *27* | 9 | 18 | *11* | 9 | 2 | Lotek Wireless Inc., LAT1400 (11x35mm, 5.2g) | Wildlife Computers Inc., SPOT275 (86x17x18mm, 35g) |
|  | GEPE | BIS | *30* | 15 | 15 | *7* | 2 | 5 |  |  |

Table S1. Location, number, and sex of Adélie (ADPE) and gentoo (GEPE) penguins sampled and tracked each year during the chick-rearing phase of the breeding season near Palmer Station, Antarctica, from 2009-2014. HUM= Humble Island, BIS= Biscoe Point, TOR= Torgersen Island. Time-depth-recorder (TDR) & satellite tag manufacturer and model included with tag specifications in parentheses.
